# Emergent Properties and Toxicological Considerations for Nanohybrid Materials in Aquatic Systems

**DOI:** 10.3390/nano4020372

**Published:** 2014-06-03

**Authors:** Navid B. Saleh, A. R. M. Nabiul Afrooz, Joseph H. Bisesi, Nirupam Aich, Jaime Plazas-Tuttle, Tara Sabo-Attwood

**Affiliations:** 1 Department of Civil, Architectural and Environmental Engineering, University of Texas at Austin, Austin, TX 78712, USA; E-Mails: navid.saleh@utexas.edu (N.B.S); rnabiul@utexas.edu (A.R.M.N.A.); nirupamaich@utexas.edu (N.A.); jplazas@utexas.edu (J.P.-T.); 2 Department of Environmental and Global Health, Center for Human and Environmental Toxicology, University of Florida, Gainesville, FL 32611, USA; E-Mail: jbisesi@phhp.ufl.edu

**Keywords:** nanohybrids, carbon, metal, nanotoxicology, ecotoxicology, aquatic

## Abstract

Conjugation of multiple nanomaterials has become the focus of recent materials development. This new material class is commonly known as nanohybrids or “horizon nanomaterials”. Conjugation of metal/metal oxides with carbonaceous nanomaterials and overcoating or doping of one metal with another have been pursued to enhance material performance and/or incorporate multifunctionality into nano-enabled devices and processes. Nanohybrids are already at use in commercialized energy, electronics and medical products, which warrant immediate attention for their safety evaluation. These conjugated ensembles likely present a new set of physicochemical properties that are unique to their individual component attributes, hence increasing uncertainty in their risk evaluation. Established toxicological testing strategies and enumerated underlying mechanisms will thus need to be re-evaluated for the assessment of these horizon materials. This review will present a critical discussion on the altered physicochemical properties of nanohybrids and analyze the validity of existing nanotoxicology data against these unique properties. The article will also propose strategies to evaluate the conjugate materials’ safety to help undertake future toxicological research on the nanohybrid material class.

## 1. Introduction

Richard Feynman’s “plenty of room at the bottom” vision inspired the synthesis and manipulation of nano-scale materials to achieve unique physical, chemical and electronic properties for a wide range of applications [1]. As commercial materials demanded higher performance and needed to display multiple functions simultaneously, materials research had to shift its focus from singular nanomaterials (NMs) to hierarchical ensembles [2,3,4]. Such nano-scale conjugates are commonly termed nanohybrids (NHs), which bring together atypical combinations of metals, metalloids and carbon-only nanostructures with unique soft and hard external coatings [2,5,6,7]. The pursuance of such ensembles has expanded the scope of applications [8,9] and resulted in a very large set of new materials with unknown environmental and health risks. Therefore, it is critical to develop an effective strategy for assessing the safety of this large and ever expanding set of NH materials.

The primary motivation of NH synthesis was to create composite materials that exhibit the enhancement of the component properties. For example, in the field of nuclear medicine, chelated radioisotopes of different metals are used despite their kinetic instability and release of toxic metals within the body [10]. Incorporation of such metals within fullerenes, known as endohedral metallofullerenes (EMFs) [5], makes the ensembles highly stable and provides an excellent alternative to the current options [5,10]. Such NHs are applied in a wide range of applications, including biomedical products and devices (e.g., nuclear medicine, drug delivery, cancer therapy [11,12]), electronic applications (e.g., nanoelectronics [13], super-semiconductors [14,15] and optoelectronics [16]), renewable energy (e.g., solar cell technologies [17,18,19], electrochemical fuel cells [3,20,21], catalysts [3,7,22]) and environmental remediation [23,24,25]. The effectiveness of conjugation thus realized continues to broaden the scope of ensemble applications, manifesting unique physical, chemical and biological properties. Such a wide application scope increases the likelihood of exposure and associated risks, as aquatic systems have a high potential for acting as a ‘sink’ for environmental contaminants. This increased production of NHs and the potential for exposure raises an obvious question: do the established nanotoxicological theories and testing strategies for aquatic systems hold true for these ensembles with emergent properties?

The field of nanotoxicology emerged from the study of particulates [26,27,28]. Historically, deleterious effects from exposure to ultrafine particles, e.g., PM_10_, PM_2.5_, as well as from mineral fibers, such as asbestos, raised safety concerns and resulted in comprehensive evaluation of their effects on environmental and human health. Particulate toxicology evolved through classical toxicity investigations utilizing both *in vivo* and *in vitro* techniques*.* Inhalation, instillation and oral exposure with endpoints of mortality, metabolic changes, biodistribution within the species body, reproductive and other physiological behavioral changes were evaluated during *in vivo* studies [29,30,31,32,33]; while cellular level studies concentrated on uptake, cell viability, DNA structural damage, immunogenicity and apoptosis [34,35]. Such detailed investigations were feasible due to a limited set of variables on the particulate side, which was severely compromised after the advent of nanotechnology. New particles with unique sizes, shapes, chemical identities and a variety of surface functionalization brought forward new challenges in evaluating the particulate-based toxicological effects of a widening set of nano-scale materials. The toxicological community has focused on revealing the mechanisms of toxicity and identifying key physicochemical properties, e.g., size, shape, surface chemistry and dissolution, that contribute to the observed toxic responses [36,37,38,39], thus narrowing down the scope of work. As the materials field moves from singular NMs to conjugated NHs, similar challenges reappear; *i.e.*, what properties should the toxicological community now be concerned about? Will the NHs elucidate properties that can be described as a summative product of the individual components, or will novel emergent properties surface due to such conjugation? These questions center on recent literature presenting evidence of the altered and novel properties of such NHs. 

Most NHs are composed of NMs with unique chemical origin or physical properties. When conjugated, the resultant properties of the ensembles are expected to be different. It is also likely that during hybridization, one or more of the components’ properties will become dominant, which can be a function of the mode of conjugation or the synthesis procedure. Such changes may be manifested in their resultant size [40], shape [41,42], crystalline structure [41,42], surface chemistry [43], dissolution properties [44], sorption characteristics [45], band-gap energetics [46], oxidation resistance [40], *etc.* Furthermore, the emergence of unique and novel properties are also likely; e.g., unique physical and mechanical behavior, enhanced reactivity and localized changes in chemical or electronic properties can emerge, which have not been highly considered as governing parameters for toxicity determination. Bimetallic core-shell NHs have been generated possessing simple spherical [47] to cubic [41], rod-like [41], plate-like [42], triangular [48] or triangular-bipyramidal [41] structures. Complex polyhedral [49], bipyramidal [50] and dumbbell [51] configurations have also resulted from hybridization. Hierarchical three-dimensional (3-D) exotic nano-structures are also obtained when exohedral conjugation occurs between zero-D fullerene, 1-D carbon nanotube and/or 2-D graphene plates [52,53].

Similarly, mechanical properties, such as stiffness, have also shown to be altered due to fullerene insertion in nanopeapods [53,54]. Moreover, bandgap modulation and variation in electronic attributes due to the insertion of fullerene onto carbon nanotubes are also observed [46]. Similarly, reactivity has been altered due to conjugation; titanium dioxide hybridized with platinum to form binary electrocatalyst has been shown to induce a strong metal support interaction (SMSI) phenomenon [55,56], resulting in altered sorption properties. The emergence of such novel properties or alterations to the known physicochemical characteristics of individual nanoparticles will most certainly affect NHs’ environmental interactions [43,57], compared to what has been observed with component materials [26,34,58,59]. However, as NH synthesis infers infinite combinations of individual NMs to obtain a large set of NHs, there is a critical need to formulate effective research strategies for assessing the environmental health and safety of this ever-expanding material class.

This paper describes the most common NHs being produced, identifies a few of their emergent properties and presents their potential implications for nanotoxicity in aquatic systems. We have focused this review on aquatic ecosystems, as with increasing use, NMs, including NHs, are likely to find their way into such environments, where they may impact relevant species. The article will introduce NH properties as per material classification and highlight key toxicity end-points for aquatic organisms and microbes, with a specific focus on metals/metal oxides and carbonaceous materials. Considerations for toxicity evaluation of NHs are described in light of the identified properties. 

## 2. Classification, Applications and Characterization of Nanohybrids

NHs can be classified on the basis of their parent materials, *i.e.*, whether the component materials are organic/inorganic or metallic/carbonaceous. For the scope of this review, NHs are mainly categorized into four classes: carbon-carbon NHs (CCNH), carbon-metal NHs (CMNH), metal-metal NHs (MMNH) and organo-metal-carbon NHs (OMCNH). Such classification is useful to identify key properties of the NHs, relevant to their safety, however, not necessarily the only basis for classifying these nano-ensembles. CCNHs are synthesized by conjugating multiple carbonaceous NMs with different geometry; *i.e*., carbon nanotubes (single-walled or multiwalled), fullerenes or graphene [52,60]. When such carbon-based NMs are combined with their metallic counterparts, the conjugates can be classified under the CMNH category [61,62,63]. Such metal and metal oxide NMs include gold [64], silver [65,66], titania [67,68], zinc oxide [69,70], alumina [71] and iron oxides [72]. Two or more metal-based NMs may also be hybridized, either by chemical attachment or by overcoating, to prepare MMNHs [73]. Moreover, synthetic macromolecules (e.g., drug molecules, proteins, dyes and other long chain polymers), enzymes and proteins are utilized to generate carbon- or metal-based NHs that can be classified under the OMCNH category. Conjugation and/or overcoating processes to generate NHs offer unique properties tailored toward a wide range of applications. Table 1 presents a summary of such applications that include: processes and devices for electronic and energy industries, biomedical applications, environmental remediation, catalytic processes, construction materials, lubrication, heat transfer and others.

**Table 1:** Types and current applications of nanohybrid materials.

Broad Application Areas	Specific Applications	NH Class	Specific Types	Citation	Environmental Exposure Pathway
Electronics and energy	Field effect transistors	CCNH	Fullerene-CNT peapods	[46,74,75]	Leachate; surface water
			Graphene-CNT hybrid	[76]	
		CMNH	Graphene-ZnO hybrid	[77]	
			Graphene nanosheet/metal nitride hybrid	[78]	
		OMCNH	Graphene-organic molecule hybrid	[79,80]	
			Poly(3-hexylthiophene)-fullerene hybrid	[81]	
	Energy storage/supercapacitors	CCNH	Graphene oxide-CNT peapods	[82]	
		CMNH	MnO_2_/CNT hybrid	[83]	
			CNT/RuO_2_ hybrid	[84]	
			Graphene-Mn_3_O_4_	[85]	
	Lithium ion batteries/storage	CCNH	Fullerene-CNT peapods	[86]	
			Graphene-CNT hybrid	[87,88,89]	
			Carbon nano-onions	[90]	
		CMNH	Graphene-TiO_2_ hybrid	[91]	
		MMNH	ZnO-Au hybrid	[92]	
	Transparent conductive films	CCNH	CNT-graphene exohedral hybrid	[76,93,94]	
			Fullerene/CNT/graphene-oxide hybrid	[95]	
		CMNH	SWNT-Au	[96]	
		MMNH OMCNH	Ag/TiO_2_ nanowire	[97]	
			Graphene-Ag nanowire	[98]	
	Photovoltaics	CCNH	Graphene-fullerene hybrid	[99,100,101,102]	
	Optical limiting devices	CMNH	CNT-fullerene	[103]	
			ZnO-graphene quantum dots	[104]	
			Graphene/TiO_2_	[105]	
		MMNH	Ag/TiO_2_ nanowire	[106]	
		OMCNH	Fullerene/CNT with porphyrins/phthalocyanines	[107]	
			dihydronaphthyl-fullerene	[108]	
		CCNH CMNH	Graphene-fullerene hybrid	[109]	
			Fullerene-CNT	[110]	
			MWNT-ZnO NH	[111]	
		MMNH	Au@TiO_2_, Au@ZrO_2_, Ag@TiO_2_, and Ag@ZrO_2_ core-shell NHs	[112]	
	Fuel Cell	OMCNH	Oligothiophene-graphene, porphyrin-graphene	[13,113]	
		MMNH	Pt-Pd	[114]	
		CCNH	Graphene-CNT exohedral hybrid	[115]	
		CMNH	CNT/TiO_2_-Pt	[116]	
			Pt-reduced graphene oxide	[21]	
		MMNH	Pd-Cu	[117]	
Biomedical	Bioimaging and cancer therapy	CMNH	Quantum dot-Fe_3_O_4_-CNT	[118]	Atmosphere
		MMNH	Au-Fe shell-core	[119,120]	
	MRI agents	CMNH	Gadofullerene	[121,122,123]	
	Drug delivery	CCNH	Fullerene-CNT	[124]	
		CMNH	Quantum dot-Fe_3_O_4_-CNT	[118]	
		MMNH	Au-Fe_3_O_4_	[125]	
		OMCNH	Pluronic F-127/graphene	[126]	
			Parclitaxel-Au	[127]	
Environmental monitoring and remediation	Chemical sensing	CCNH	Carbon nanotube-graphene nanosheet hybrid	[128]	Leachate
		CMNH	Pt-graphene	[129]	
			MWNT-zerovalent iron	[130]	
			Graphene-iron	[131]	
			Graphene-ZnO	[132]	
		MMNH	Au-Ag	[133]	
			Pt/TiO_2_ nanotube	[134]	
		OMCNH	Hematoporphyrin-ZnO	[135]	
	Biosensors	CCNH	Reduced graphene oxide-MWNT	[136]	
	Gas sensors	CCNH	Graphene-CNT hybrid	[137]	
	Contaminant degradation	CMNH	CNT-TiO_2_	[138]	
			ZnO-reduced graphene oxide	[139]	
	Pathogen detection	MMNH	Fe_3_O_4_-Au-Fe_3_O_4_ nanodumbbelland Fe_3_O_4_-AuNR nanonecklace	[140]	
			Au-Ag	[141]	
	Antimicrobial	CMNH	CdSe-Au	[142]	
			Graphene-ZnO	[132]	
			Ag-graphene oxide	[143]	
	Heavy metal removal	CCNH	Carbon nano-onions	[144]	
	Bio-imaging	CCNH	Carbon nano-onions	[145]	
Catalysis	Catalyst support/catalyst	OMCNH	CNT-enzyme	[146]	Atmosphere; leachate
		CCNH	N-doped CNT-graphene peapods	[147]	
		CMNH	CNT/Pd	[148]	
			Graphene-Au	[149]	
		MMNH	Au-Pd core-shell structure	[150]	
Construction industry	Nano-reinforcement in composites		Pt/Pd-Fe/TiO_2_	[114]	leachate
		CCNH	CNT-Graphene nanoplatelet hybrid	[151]	
	Structural health monitoring	CCNH	CNT-graphene nanoplatelet hybrid	[152]	
Miscellaneous	Antimicrobial coating/paint	CCNH	Carbon nano-onions	[153]	Leachate
	Temperature sensor	CCNH	Azafullerene-CNT peapods	[154]	-
	Heat transfer	CCNH	Graphene wrapped MWNT	[155]	-

Abbreviations: CCNH, carbon-carbon nanohybrid (NH); CMNH, carbon-metal NH; OMCNH, organo-metal-carbon NH; MMNH, metal-metal NH; CNT, carbon nanotubes; SWNT, single walled carbon nanotubes; MWNT, multi walled carbon nanotubes.

Such a widespread application of NHs necessitates a careful evaluation of their toxicity to aquatic organisms. Systematic evaluation of NM toxicity requires detailed physicochemical characterization. Most characterization techniques utilized for singular NMs are also applicable to NHs [156]. Key physicochemical parameters that are relevant to toxicity include: particle size distribution, morphology, surface potential, wettability, concentration, the presence of functional groups, adsorption properties, band gap energetics, reactive oxygen species (ROS) generation, and metal dissolution. These parameters are characterized using electron microscopy, e.g., transmission, scanning and scanning tunneling electron microscopy; several spectroscopic techniques, UV-Visible, atomic absorption, Raman, Fourier transformed infrared, X-ray photoelectron, and energy dispersive spectroscopy; interfacial characterization tools, e.g., electrophoretic mobility and dynamic and static light scattering; thermo-gravimetric analysis and inductively coupled plasma mass spectroscopy for metal dissolution. However, hybridization will likely alter some of these inherent properties or present emergent novel properties that are not typically manifested by singular NMs. Thus, it is to be ensured that classical characterization techniques utilized for singular NM characterization are appropriately adjusted to measure altered and emergent properties presented by these conjugated NHs. 

## 3. Key Properties Relevant to Toxicity

The near-atomic size factor of NMs enhances their interaction with biological species, organs, tissues and cells and has manifested unique toxicological responses [26,157]. Moreover, due to their large surface area to volume ratio, NMs provide reactive surfaces influencing particle-particle and cell-particle interactions [26,27]. Unique toxicological responses from NMs have been correlated to their geometry, chemical composition, chemical stability, and surface chemistry [26,27]. In addition to these common determinants, the release of dissolved ionic species from metallic NMs are also known to impart biological stress [158]. This section identifies potential alterations to well-known NM physicochemical attributes and the manifestation of novel emergent properties resulting from hybridization that will likely influence the toxicological outcomes of NHs.

### 3.1. Alteration of Well-Known NM Properties Relevant to Toxicity

#### 3.1.1. Bandgap Energetics, Photocatalytic Activity and ROS Generation Potential

**Figure 1:**
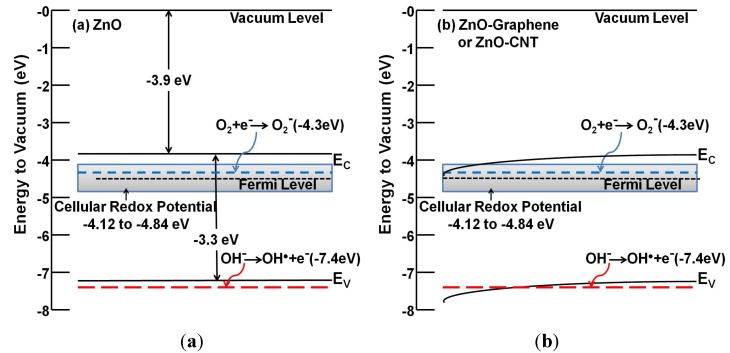
Bandgap energetics diagram of (**a**) ZnO and (**b**) ZnO-graphene or ZnO-CNT NH. The diagrams also show the relative energetic positions of the cellular redox potential (−4.12 to −4.84 eV) and relevant oxygen species (superoxides and hydroxy radicals).

Cellular toxicity resulting from exposure to carbonaceous and metallic NMs is known to be mediated by oxygen-containing radicals, such as peroxides and singlet-oxygen, collectively known as reactive oxygen species or ROS [159]. ROS generation depends on the photo- and catalytic-activity of NMs dictated by their electronic properties [160]. A ROS-mediated toxicity paradigm of metal oxide NMs hypothesizes that a surface’s ability to generate oxidative stress is directly correlated to the energetic positioning of its conduction band (E_c_) with respect to cellular redox potential [161,162]. When the E_c_ overlaps with the cellular redox potential, the nanomaterials quench the reducing capacity of antioxidants present in the cell, such as glutathione. NM hybridization significantly alters the electronic properties through band bending (Figure 1) and, thus, can display altered cellular toxicity.

One of the key reasons to design and develop NHs is to tune the bandgap for achieving the desired electronic and photoactive properties for applications in solid-state electronics [46] or pollutant degradation [63]. However, differences in the mode and extent of hybridization and the materials utilized can contribute to the differences in band-structure alterations, which can subsequently cause uncertainty in the shift of conduction band (E_c_) positioning. Such an E_c_ shift can cause overlap with the cellular redox potential and can mediate ROS generation ability. Figure 1 shows the bandgap energy diagram of (a) ZnO NM and (b) ZnO-graphene or ZnO-CNT NH. E_c_ for ZnO is positioned at −3.9 eV (Figure 1a), which resides outside the cellular redox potential range of −4.12 eV to –4.84 eV [161]. When conjugated with carbonaceous nanomaterials, e.g., CNTs or graphene, the excellent charge transfer and separation characteristics of carbonaceous nanomaterial causes band-bending of ZnO toward the CNT/graphene work function (−4.3 to −5.0 eV), as shown in Figure 1b [163,164]. Such band-edge movement causes the overlap of the E_c_ position with the cellular redox potential, thus likely causing increased photocatalytic activity, ROS generation and cytotoxicity from ZnO-graphene NHs [132,165]. Therefore, systematic evaluation of the altered band structure can hold the key to precise understanding of the ROS generation potential and the corresponding nanotoxicity.

#### 3.1.2. Dissolution Characteristics

Metal dissolution from metallic (e.g., Ag) and metal oxide (e.g., ZnO) NMs is also well-known to affect toxicity towards microbes [166], aquatic invertebrates and vertebrates [167] and higher trophic level species [168]. Hybridization of metal NMs can alter dissolution properties and, thus, can impact toxicological consequences. For example, dissolution of highly reactive Ag is reduced if protected by a thin layer of relatively inert gold (Au), irrespective of the Ag:Au ratio in the NH [44]. On the contrary, rapid dissolution of Ag is observed under physiological conditions if it surrounds an Au core [169]. Similarly, conjugation of carbonaceous NMs with metallic ones may change their dissolution chemistry, as observed in the case of Ag dissolution, where the rate of Ag^+^ ion production is decreased (leading to long-term antimicrobial actions) when conjugated with graphene nanosheets using polymeric linkers [170]. Thus, variability in dissolution properties introduced via the hybridization of NMs will likely influence nanotoxicological responses in aquatic organisms.

#### 3.1.3. Surface Chemistry

NMs’ surface functionality and chemistry control their environmental toxicity on the basis of their sorption or reactivity with the cell-membrane proteins, lipids or polysaccharides [171,172]. When NMs are hybridized, the surface characteristics become altered due to the incorporation of functional moieties, solvent effects, surface coating, bonding characteristics and linking molecules that conjugate multiple nanomaterials [156] and will likely influence NH-cell interaction. For example, nanopeapods that encapsulate fullerenes within SWNT cylindrical structures may prevent the direct interaction of fullerenes with cells or aquatic species, whereas exohedral fullerene-CNT NHs or nanobuds will likely present both CNT and fullerene surfaces to biological species, modulating toxicological responses in a more profound way [52,156]. The chemistry and nature of bonding between fullerenes and CNTs will also play a significant role in the toxicity of the NHs. Seamless conjugation of fullerene-CNT by covalent bonding produces less soluble hybrids (*i.e.*, nanobuds) than those conjugated via organic linkers, creating non-covalent bonds between CNT and fullerenes [156]. This difference in solvent affinity will have implications in hydrophobic interactions during NH-cell interactions. Additionally, organic-carbonaceous hybrids, such as MWNT-porphyrin conjugates, introduce toxic moieties that can increase antimicrobial effects (when compared to pristine MWNT), via ROS-mediated cell damage under visible irradiation [173]. Thus, the presence of multiple reactive surfaces with changes in bond structure between multiple NMs, as well as the presence of unique inorganic/organic moieties will influence NH toxicity.

### 3.2. Emergence of Novel Toxicological Properties for NHs

#### 3.2.1. Dimensionality and Surface Morphology

NM shape and size have been established as key physicochemical parameters for their toxicological responses enabled by their underlying surface area and morphological effects [36,174,175,176]. However, these properties are dependent on the dimensionality of the material; *i.e.*, whether it is zero-dimensional (fullerenes or spherical metals), one-dimensional (CNTs or metal nanorods) or two-dimensional (graphene or plate-like metal NPs). For example, CNTs’ needle-like appearance can induce increased toxicity compared to the globular or planar structures of fullerenes or graphene, respectively [174,175]. However, when these NMs are conjugated, altered dimensionality is the most obvious consequence, dictated by the types of parent material and the mode of conjugation. For example, when fullerenes or graphene are incorporated endohedrally within the hollow structure of a CNT, e.g., nano-peapods, the one-dimensional CNTs will likely mask the zero or two-dimensionality of the fullerenes [177] or graphene [147]. Furthermore, exohedral or outer surface conjugation of carbon-based nanostructures can result in unique three-dimensional structures, as observed in the case of nanobuds [52,178], other hierarchical configurations (e.g., grapevine-like fullerene-CNT NHs [103], or multilayered CNT-graphene NH [89] structures). The role of dimensionality here can be realized as the presentation of the altered geometry of the NHs to cells and tissues. For example, CNTs’ well-known asbestos-like [179] or fullerenes’ ROS-dependent toxicity [180] might be further reinforced by membrane disruption dynamics, due to the edge roughness of graphene [181] when conjugated as a single NH unit. Similarly, metal NMs when decorated on 2-D graphene or 1-D CNT surfaces can lead to less agglomeration, resulting in increased available reactive surface area and, thus, can alter cellular interactions. Moreover, polydispersity and different shapes of metal NMs (from cubic or spherical to dumbbell shaped or flowerlike structures) conjugated on the CNT/graphene surfaces will generate a wide-array of diverse surface morphologies, possibly modulating the cytotoxicity of these unique NHs. Thus, dimensionality can serve as one of the emergent parameters, causing unpredictability in biological responses from NH exposures. 

#### 3.2.2. Mechanical Properties

Hybridization of NMs, particularly CNTs with other structures, affects their mechanical properties; *i.e.*, mechanical stiffness, bendability/curling ability, *etc.* CNTs are known to have excellent compressive, tensile and flexural strength [182]. Encapsulation of fullerenes has been shown to increase the bending strength of SWNTs [183], resulting in stiffer tubules [54]. On the contrary, the elastic moduli of graphene nanosheets are predicted to decrease, due to fullerene conjugation on the planar surfaces when estimated via molecular dynamics simulations [184]. On the other hand, graphene exhibits enhanced mechanical properties when decorated with AgNPs, displaying increased tensile strength and a Young’s modulus by 82% and 136%, respectively [185]. Such evidence indicates that NH mechanical properties will likely be different than their parent materials; such emergent behavior may lead to novel physical interactions, as well as unique particle-particle interactions during exposure to biological organisms. 

#### 3.2.3. Synergistic Properties

One of the key underlying reasons for conjugation or hybridization is to acquire synergy between multiple functionalities of NMs; *i.e.*, the conjugation of two or more NMs manifests enhanced- or multi-functionality, which otherwise may not be attainable. Therefore, removing one component from the NH will compromise the synergy between multiple properties. For example, unique hierarchical structures, like Ag-supported-graphene-wrapped-ZnO (Ag-graphene-ZnO NH), can display synergistic functionality. Photoactive ZnO produces charges (*i.e.*, electron-hole pairs) under illumination, while highly conductive graphene helps to increase the degree of charge separation and prevent recombination and Ag acts as an electron sink, improving the photodynamic degradation of pollutants [186]. While synergism in NHs may be beneficial for applications, its contribution towards toxicity and cellular interaction should not be ignored. For example, combinatory biocidal and photocatalytic activity in the dark and illuminated conditions is observed in Ag-TiO_2_ core-shell formulations [187]. In this case, TiO_2_ not only protects the AgNPs from fast dissolution (long-term antimicrobial actions), but also serves as an AgNP carrier to bacterial entities. Moreover, Ag^+^ dissolution accompanied by the evolution of ROS from photoactive TiO_2_ presents synergism in combined bactericidal performance [188]. Such properties that work in sync are unique to this horizon NH material class and necessitate a fundamental understanding of their effects on aquatic species. 

**Figure 2:**
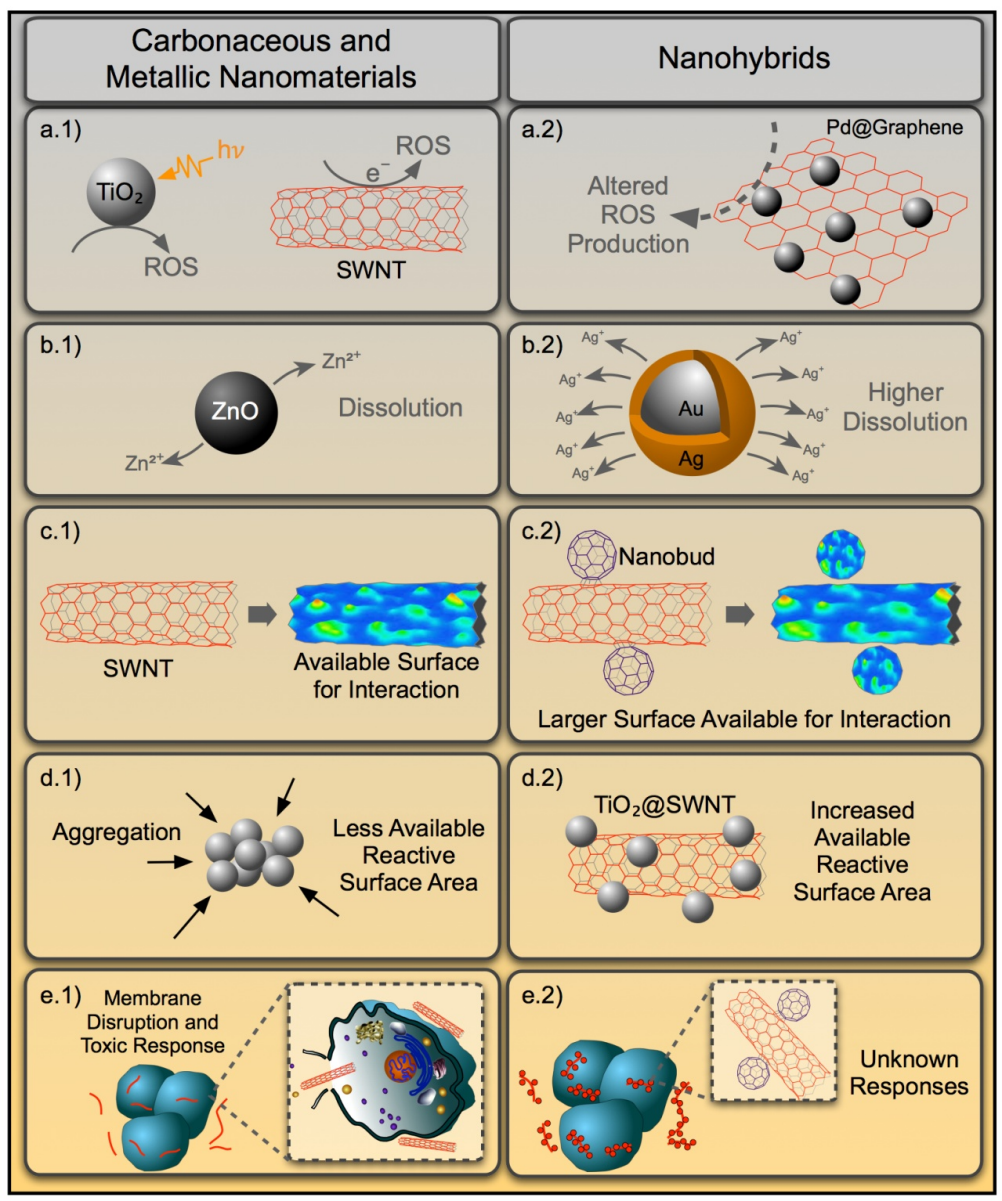
Diagram showing the relevant properties of carbonaceous and metal NMs that are associated with toxicity (**right panels**, **a.1**–**e.1**). How these properties might be altered for nanohybrid materials is displayed in the corresponding left panels (**a.2**–**e.2**).

## 4. Toxicological Implications for NHs Based on Current Biological Effects and Mechanisms of Action

### 4.1. Aquatic Nanoparticle Toxicity Testing Strategies

Compared to assessments on mammalian models, fewer studies have investigated the impact of NHs on the health of aquatic organisms to try and understand the toxicity and modes of action of singular NMs. It is only prudent to assess the potential implications from NHs by evaluating component material impacts. It is to be noted, however, that due to the sheer diversity in NM types and associated variables (metal content, purity, size, shape, surface chemistry) and combinations, the toxicity data can be quite challenging to consolidate and interpret for a given NH [189]. Two types of singular NMs that have been a prominent focus of study in aquatic toxicology are metal- and carbon-based NMs. For metals, most studies have focused on TiO_2_ [190], Ag [191], ZnO [31] and CuO [192] nanoparticles, with a smaller subset examining quantum dots [193], Al [194], Ni [195], Ce [196], Fe [197] and Au [198] particles. The carbonaceous materials primarily investigated include multiple types of carbon nanotubes (*i.e.*, SWNTs *vs.* MWNTs) [199], graphene [200] and fullerenes [201]. Possible toxicological implications for hybridization of carbonaceous and metallic NMs to make different NHs have been summarized in Figure 2. Detailed description of such properties' alteration has been provided in the previous section. In this section, we review what has been learned with regard to the biological effects and mechanisms of action (MOA) in aquatic species exposed to these select types of singular NMs. We additionally discuss the application of such knowledge to NH toxicity testing in light of the potential for NHs to possess new emergent properties.

In the field of aquatic toxicology, traditional animal models of study found throughout the literature include pelagic invertebrates (*Daphnia sp.*), benthic invertebrates (*Hyalella azteca*), large (*i.e.*, Rainbow trout) and small (*i.e.*, fathead minnow, *P. promelas*) fishes and a number of algae species [202]. Researchers have also utilized models that allow for the testing of specific endpoints (such as development in zebrafish, *Daniorerio* [203]) or species-specific effects on marine algae and invertebrates [204], plants [205], emergent insects [206], filter feeding organisms [207] and snails [208], among others. As each nanoparticle has its own set of unique properties, as discussed above, the selection of an appropriate test organism and exposure route can maximize our understanding of the potential effects. This can pose quite a challenge, as we have a poor understanding of which NMs and in what concentrations they will be found in the aquatic environment.

### 4.2. Biological Mechanisms of Metal/Metal Oxide Nanoparticle Toxicity

While information about the acute and chronic overt toxicity of metal nanoparticles is important for understanding the risk these materials pose, elucidation of the underlying mechanisms of toxicity may help us mitigate these risks more effectively. Studies on these mechanisms have proposed a diverse array of potential targets. The most widely probed mechanism is oxidative stress as a result of the production ROS, as discussed earlier. While internal ROS production is a natural phenomenon in biological tissues, excessive ROS (from external sources) can cause oxidative damage and cell apoptosis. Exposure to TiO_2_, Ag, Zn and Al nanoparticles have all been shown to increase ROS by direct measurement in tissues of exposed organisms [203,209,210,211]. The measurement of lipid peroxidation (LPO) can also serve as an indicator of oxidative stress, as ROS can damage the lipid bilayer of cell membranes. Examples of metal nanoparticles increasing LPO include numerous fish species exposed to ZnO and TiO_2_ [212,213,214].

Plants and animals have natural defense systems to mitigate the effects of oxidative stress. Numerous antioxidants are produced to scavenge ROS and prevent potential damage, including super oxide dismutase (SOD), peroxidase, catalase (CAT) and glutathione (GSH). Increased levels of these antioxidants have been used as biomarkers of oxidative stress in numerous studies, including tilapia exposed to Ag nanoparticles [215], carp exposed to ZnO [214], marine invertebrates exposed to CuO [204] and *Daphnia magna* exposed to TiO_2_ [216]. Measurement of the phase II enzyme responsible for the metabolism of GSH, glutathione s-transferase (GST), has also been used as a downstream indicator of oxidative stress [204,212]. Metallothionein, a protein involved in binding free metal and decreasing oxidative stress, has also been shown to be upregulated during metal nanoparticle exposure [217,218]. In addition to acting as biomarkers of exposure, disruption of natural antioxidant levels has also been proposed as a mechanism of toxicity. For example, *Daphnia magna* exposed to Cu nanoparticles exhibited an initial increase in antioxidants to combat oxidative stress, but over time, antioxidant production decreased, potentially due to extensive damage [219]. This phenomenon was also seen in the livers of fish exposed to TiO_2_ [212].

The measurement of oxidative stress biomarkers provides strong evidence of metal nanoparticle exposure, but the measurement of downstream effects can provide biomarkers of the effect for these materials. Studies have shown that TiO_2_, Ni and Ag nanoparticles caused membrane breakage, leading to decreased membrane integrity [195,209,220]. Differential production of Na/K ATPase during nanoparticle exposure may also be indicative of the loss of cell homeostasis [213,221,222]. Irritation of the gills of zebrafish by Ag and Cu nanoparticles led to increased gill filament width, which could cause respiratory distress [192,223]. Though a large majority of mechanistic studies have focused on the mechanisms of toxicity in animals, a few researchers have examined toxic mechanisms in plants and phytoplankton. In addition to some of the biomarkers of exposure described above, depletion of chlorophyll content and decreased photosystem II activity are commonly measured biomarkers of the effect as a result of exposure to Ag, Cu, Zn and Cu nanoparticles [224,225,226]. 

It is important to note that many of the properties of metal-based NMs that cause oxidative damage to aquatic species make these materials valuable for manufacturers as antimicrobial agents [227]. However, these properties may have unintended consequences in bacterial communities found in our wastewater treatment plants and aquatic ecosystems [228]. ROS production in particular has been shown to cause general oxidative stress in a number of bacterial species, as well as lipid and protein oxidation, DNA/RNA damage and the interruption of cell signaling pathways [229].

Perhaps the most difficult aspect of examining the aquatic toxicity of metal nanoparticles is assessing the contributions of dissolved metals from the particles, and the particles themselves have on toxicity. Many studies have attributed the toxicity of metal nanoparticles to the dissolved fraction [38,191], while others hypothesize that particles and dissolved ions may have individual and potentially different, toxic mechanisms [192,230]. The mechanical separation of intact particles from dissolved ions allows for researchers to examine the individual contribution of each metal species on toxicity [231]. For example, Griffit *et al.* [192] exposed zebrafish to Cu nanoparticles, as well as ionic copper. The dissolved fraction of copper was not enough to explain the toxicity of the copper nanoparticles, and gene expression profiles between the two exposure scenarios showed differential modes of action, suggesting that nano-copper toxicity involves more than just dissolved copper toxicity.

Though intact nanoparticles and their dissolved ions may have individual contributions to toxicity, it is also possible for metal nanoparticles to act as catalysts for the increased toxicity of different metals. Wang *et al.* 2011 [232] showed that the combination of arsenic (As) with TiO_2_ particles caused higher toxicity than equivalent individual exposures, with a similar response seen in the presence of Al particles [222]. Researchers have shown that metals can bind to TiO_2_, which may facilitate increased internalization and, therefore, increased toxicity [221,233].

While direct interactions of metal nanoparticles and their dissolved ions with biochemical targets present numerous toxic mechanisms, physical interactions with organisms may indirectly cause toxicity. For example, TiO_2_ has been implicated in decreasing growth and reproduction in *Daphnia magna*, due to changing gut morphology and clogging the gut, which may, in turn, decrease nutrient uptake [234,235]. Ag nanoparticles also decreased food intake and digestion in snails, suggesting digestive damage [236]. Direct binding of TiO_2_, Ag and Ce nanoparticles to *Daphnia magna* carapace has also been hypothesized to interfere with normal molting activity [196,237]. Exposure to metal oxide nanoparticles has also been shown to decrease hatching success in zebrafish embryos [230,238]. One possible explanation is that dissolved ions from the particles freely diffuse through the embryonic chorion and chelate the enzyme responsible for breaking down the chorion (zebrafish hatching enzyme, ZHE1), rendering it inactive. As a result, larval fish cannot hatch and eventually die [239,240].

All of the above mechanisms may also be influenced by the presence or absent of numerous abiotic factors that likely influence the toxicity of metal NMs in aquatic systems, which include salinity, pH, temperature and the presence of divalent ions [202]. However, one factor that may have the greatest influence on NM toxicity is the presence of dissolved organic matter (DOM). This factor becomes key during NM transformation in the environment. For metal NMs, DOM in aquatic systems has been shown to influence both size and toxicity. In general, DOM helps to stabilize particles, effectively decreasing particle size and aggregation [241,242,243], and as a result, toxicity is typically reduced in the presence of DOM. This has been demonstrated for the metal NMs, Ag and TiO_2_ [241,242,244,245].

The influence of natural light on particle toxicity also cannot be ignored if toxicity tests are to represent environmentally realistic exposures, a property especially important for assessing metal toxicity. Select metal nanoparticles have been shown to have increased and decreased toxicity in the presence of UV light. TiO_2_ exhibits strong photoreactivity by the formation of reactive oxygen species in the presence of ultraviolet light [246]. While this property may be favorable for applications, such as anti-microbial coatings, the photocatalysis of TiO_2_ has been shown to increase toxicity in aquatic organisms [242,243,247]. UV light has also been shown to influence particle size and resulting toxicity, though results have been contradictory. Poda *et al.* [241] showed that UV lights caused the oxidation of PVP coatings on silver nanoparticles, which, in turn, caused a decreased particle size and increased dissolution, while Shi *et al*. [248] showed that sunlight caused an increased particle size and particulate deposition. In both cases, the addition of UV light decreases the toxicity of Ag particles, though the hypothesized mechanisms differed.

### 4.3. Biological Mechanisms of Carbon Nanoparticle Toxicity

Current research centered on the toxicity of carbon NMs in aquatic organisms has been minimal compared to mammalian systems, where there has been a heavy emphasis on carbon nanotubes and their pulmonary exposures and effects [249]. The primary carbon NMs that have been studied in aquatic organisms include SWNTs, MWNTs, fullerenes and graphene. The toxic effects of these materials have been evaluated in a number of species, including microbes, invertebrates (daphnia, snails, mussels) and a number of fishes. Similar to metal NMs, the primary biological MOA that has been most commonly evaluated for these materials is oxidative stress, which is typically assessed in concert with mortality, survival, growth and reproduction. However, this mechanism has not been met without controversy, as some reports have emerged that suggest organisms can manage oxidative stress and that other more subtle MOAs should be considered. It has also been proposed that the chemicals used to suspend carbon NMs (*i.e.*, surfactants) may be a significant contributing factor to oxidative-mediated mechanisms [250]. A few studies have additionally investigated impacts on cell membrane integrity and immune parameters, such as the modulation of the expression of genes, IL-1β and INFα, in trout macrophage primary cells exposed to carbon nanotubes [251]. 

Understanding whether NMs are absorbed and distributed in biological systems is critical in defining toxicity and setting regulatory standards (*i.e.*, limits of exposure). In general, this has been difficult to assess for carbonaceous nanoparticles, as we have limited availability of adequate analytical detection methods (reviewed by Edgington *et al.* [252]), which have made such assessments difficult. Due to the difficultly in tracking, detection and quantification of carbon NMs in aquatic organisms, most reports have relied on radiolabeled materials. Of the few studies performed to date, most agree that carbon nanomaterials are not readily absorbed into organisms through a dietary route. For example, separate studies collectively show that CNTs enter the gut of invertebrates (copepods, *Daphnia*), where they either accumulate or are eliminated with no evidence that they are absorbed across the gut epithelium [253,254,255,256]. More recent work by our group has utilized near-infrared fluorescence (NIRF) imaging and quantitative methods to show similar results in fathead minnows exposed to SWNT by gavage [255].

Investigations of carbon nanoparticle toxicity have focused on a number of properties inherent to the particles, including shape and surface functionalization. Much of our understanding of shape effects comes from studies performed in mammalian systems, which show that the fibrous tube-like shape of CNT makes it easy for them to penetrate cell membranes. However, the majority of studies performed in aquatic organisms do not support that carbon nanomaterials are taken up into tissues. Only a few studies have performed comparisons of graphene and/or graphite and CNT in efforts to investigate shape effects on biological effects. For example, Kang *et al.* [257] tested the ability of CNT, nC_60_ and graphite to cause toxicity to multiple bacterial strains in wastewater effluent and driver water extracts. Results of this work reveal that graphite and C_60_ were less toxic, as measured by cell inactivation, compared to the SWNT. While a number of parameters likely contribute to this observation, the diverse shape of the particles should be considered as a contributing factor. Conversely, other studies have shown that graphene nanosheets with sharp edges cause considerable damage to bacterial cell membranes [258], implying that shape has an important influence on toxicity for some species. 

One property that makes carbon nanomaterials of the same type (*i.e.*, CNT) potentially distinct from a toxicological point of view is their surface chemistry. This property has been more widely studied compared to the influence of shape in aquatic models. Variations in surface chemistry can be tightly controlled during particle synthesis and include such variations as the addition of neutral, positive or negative functional groups. The impacts of such diverse surface modifications of CNT were shown in studies on *Daphnia*, where carboxylation of SWNTs increased toxicity, while functionalization with amine groups or poly-ethylene glycol (PEG) decreased toxicity. In the same study, the exposure of *Daphnia* to unfunctionalized fullerenes was associated with decreased reproduction and growth, which improved when the fullerenes were hydroxylated [259]. Interestingly, in a follow-up study, this same group showed that the toxicity profiles for aminated SWNT were not consistent in multigenerational effects. In fact, these SWNTs decreased survival or reproduction in F_1_ and F_2_ generation *Daphnids* [260].

Despite the lack of observed acute toxicity, absorption and systemic distribution, carbon NMs may pose more subtle health effects. Current theories of study include the ‘Trojan horse hypothesis’, which suggests that due to their sorptive nature, carbon NMs can interact with other more well-known chemical contaminants, which can then be transported into organisms through multiple means. While this hypothesis is being widely investigated in the drug delivery arena, few studies have been conducted that are relevant to aquatic systems. Conversely, the interaction of carbon NMs with such contaminants may limit their bioavailability, resulting in decreased toxicity. While a number of studies support the ability of CNTs, fullerenes and graphene to sorb well-known toxic chemicals, such as polychlorinated biphenyls (PCBs), polycyclic aromatic hydrocarbons (PAHs) and xenoestrogens [261], only a few studies have probed the associated toxicity. In a study by Parks *et al.* [262], the sorption of SWNTs with PCBs was shown to decrease bioaccumulation and toxicity to benthic organisms. Similar results were observed in zebrafish hepatocytes co-exposed to fullerenes (C_60_) and metalloid arsenic (As(III)), where the overall As toxicity, as measured by oxidative stress, was diminished by the presence of C_60_ [263]. Furthermore, due to their sorptive nature, carbon NMs may produce a state of ‘nutrient depletion’ as an indirect mechanism of toxicity [264,265,266], as they sequester growth factors and other molecules necessary for good nutrition. It has been observed in mammalian studies that due to the sorptive nature of CNT, they are able to interact with proteins, growth factors and other molecules; however, this hypothesis has not been examined in aquatic models.

Similar to metals, the presence of DOM has been shown to influence both environmental fate and transport, as well as the toxicity of carbonaceous nanomaterials. A number of studies report that altering the pH varies the surface charge of carbon nanotube, which alters the way DOM coats the particles [267,268]. In essence, this ultimately changes particle stability in aqueous environments, typically resulting in increased aggregate size. Interestingly, DOM-suspended MWNT did indeed show increased aggregates; however, this modification of the MWNT did not result in an altered toxicity profile compared to non-DOM MWNT suspensions [267]. Conversely, studies performed on Japanese medaka (*Oryzias* *latipes*) embryos show that while the toxic effects of Aqu/nC_60_ and raw MWNT were not altered with the addition of DOM, the adverse effects associated with exposure to nC_60_ (prepared in toluene) and acid-treated MWNTs were reduced [269]. A reduction in microbial toxicity has also been observed in microbes exposed to nC_60_ in the presence of DOM [270].

## 5. Application of Biological Effects of Constituent NMs to Understanding NH Toxicity

In assessing the health impacts of NMs, there has been a movement to understand the key properties of nanoparticles and the environmental influences that drive adverse health outcomes. As described above in the previous sections, studies have been heavily focused on the influence of DOM, dissolution (for metals), surface chemistry, shape and their relationship to oxidative stress, adsorption and gross endpoints, such as mortality and reproduction. While these studies provide important toxicological data for single NMs, this information may not be entirely applicable to predicting and understanding the potential toxicity of NHs. Hybridization of NMs will likely influence the toxicological responses of aquatic organisms. A focus of current testing strategies thus far has centered on correlating key attributes of NMs to select biological end-points. Thus, it is expected that the alteration of such toxicologically relevant properties or the introduction of novel emergent ones will significantly influence the potential health and ecosystem impacts of NHs. Oxidative stress imparted toward microbes or aquatic organisms is highly dependent on generated ROS and their specific types; *i.e.*, superoxides, hydroxyl radicals or singlet oxygen. Thus, it follows that hybridization-induced changes in band-gap energetics that influence ROS generation will likely have an impact on toxic effects that include signal transduction, DNA damage, lipid peroxidation, enzyme dysfunction, mitochondrial oxidative disorder and apoptosis. In fact, changes in ROS production and subsequent toxicity implications of Fe NP for overcoated structures, *i.e.*, for Fe/Ni, Fe/Pt, Fe/Pd and Fe/Cu, have already been reported in the literature [271]. As materials science communities continue to control band gap energetics through the production of NHs, particularly the conjugation of multiple metal/metal oxides or with carbonaceous surfaces, it will be important to assess altered ROS production by such novel materials and their impacts on aquatic species.

Similarly, dissolution products of NMs, e.g., from ZnO, CuO or Ag, can damage cell membranes via reaction with transport proteins, produce chelating compounds with essential intracellular proteins or alter cellular metal ion concentrations, resulting in organelle damage. These cellular consequences are highly dependent on the dissolved metal speciation and ion concentration. When hybridized, metal dissolution rates will likely become altered, either due to over-coating with a different metal of varying solubility or due to the increased surface area of metal NMs as a result of their controlled distribution over a secondary surface. A recent study showed the comparative toxicity of Au-Ag hybrids towards *Daphnia magna* in the presence of synthetic surface waters (SSF) and presented LC_50_ values for two Au-Ag combinations [44]. The displayed toxic effects from these NHs lay in between the manifested effects from singular AgNPs and AuNPs. Similar responses are likely to occur from overcoated NHs, as well as from exohedrally or endohedrally distributed metal/metal oxide-carbonaceous NHs. However, significant uncertainty remains regarding how these overcoated and conjugated NHs will manifest dissolution properties that can be linked with toxic responses.

Biological responses associated with exposure to NMs may also be influenced by shape and size. Conjugation will inherently introduce new dimensional changes, bringing forward NMs with altered size/shape, surface area and reactivity [272,273]. This emergent dimensionality attribute will likely influence NH toxicity by altering interactions with cellular membranes, which may alter the mechanisms of particle uptake, either by diffusion or energy-requiring processes. Furthermore, placing one NM of a certain density onto a second one may also change the mechanical stiffness of the NHs. An abundance of literature already reports the influence of stiffness/rigidity on the cytotoxicity for singular NMs [274,275,276]. In addition, adverse biological effects of other ‘stiff’ particles, which are not easily cleared, such as crocidolite asbestos fibers, are well documented. Such altered mechanical property may lead to significant changes in NM clearance from biological tissues and organisms. Both changes in dimensionality and stiffness may thus lead to changes in the way these NHs interact with biological molecules, which is an MOA being highly studied for carbon-based nanoparticles.

## 6. Conclusions

Materials science has moved on from singular NM synthesis and functionalization to hierarchical ensembles of more complex NHs. Multifunctionality is almost a necessity for many current and future applications. Thus, NHs will be the future of nano-scale materials that synergize multiple functions. It is likely that hybridization will lead to the alteration of existing properties or the emergence of properties not yet characterized that need to be considered in assessing toxicity in aquatic systems. As we are still beginning to define such properties of NHs, we should consider how toxicity might be altered as a result. As a research community, we should strive to develop standard protocols for accurate measurements of these properties, perform systematic studies to assess variations of such properties and bridge our understanding of these properties to underlying cellular mechanisms of action. Since the production of such an ever-expanding set of NHs with new compositions and emergent properties is imminent, the evaluation of their biological behavior is necessitated.
